# Structural Inequities and Barriers to Accessing Kidney Healthcare Services in the United States: A Focus on Uninsured and Undocumented Children and Young Adults

**DOI:** 10.3389/fped.2022.833611

**Published:** 2022-04-05

**Authors:** Franca M. Iorember, Oluwatoyin F. Bamgbola

**Affiliations:** ^1^Division of Pediatric Nephrology, Baylor College of Medicine, Children's Hospital of San Antonio, San Antonio, TX, United States; ^2^Division of Pediatric Nephrology, SUNY Downstate Medical Center, Brooklyn, NY, United States

**Keywords:** structural inequities, racial disparities, uninsured, insurance coverage, chronic kidney disease, immigration

## Abstract

The population of children living in poverty and lacking healthcare insurance has increased in the United States of America in the last decade. Several factors have been responsible for this trend including illegal immigration, socioeconomic deprivation, young age, racial segregation, environmental degradation, and discriminatory housing policies. These systemic barriers have contributed to the exclusion of families from essential healthcare services. They are also contributory to the development of chronic illnesses (such as dialysis-dependent kidney disease) that are debilitating and frequently require considerable therapeutic resources. This unfortunate scenario creates a never-ending vicious cycle of poverty and diseases in a segment of society. For pediatric nephrologists, the challenges of caring for uninsured children with chronic kidney disease are all too familiar. Federally funded healthcare programs do not cover this patient population, leaving them the option of seeking care in emergency healthcare settings. Presentation with a critical illness often necessitates urgent placement of vascular catheters and the choice of acute hemodialysis. Adverse social environment influences the need for protracted chronic hemodialysis and a delay in kidney transplantation. Consequently, there is greater comorbidity, recurrent hospitalization, and a higher mortality rate. New policies should address the deficit in health insurance coverage while promoting social programs that will remove structural barriers to health care resources for undocumented children and young adults.

## Introduction

The last decade has seen an increase in the number of people including children and young adults living in poverty and consequently without health insurance coverage in the United States of America. A report released by the United States Census Bureau in September 2020 showed a 1% increase in the number of persons living in poverty between the years 2019 and 2020. The largest increase was seen in the Black and Hispanic populations (1). Similarly, there was a 0.1-point increase in the number of uninsured people from 2018 to 2020. Amongst the poor, the percentage of uninsured children, rose from 7.8% in 2018 to 9.3% in 2020. The largest increase was again seen in Black, Hispanic, foreign-born and non-citizen children (1). In a report by the Georgetown Center for Children and Families, there was an increase of more than 400,000 in the number of uninsured children between 2016 and 2018, with the largest increase observed in Latino and White children (2). Access to specialty healthcare has been shown to be directly linked to health insurance status of children, with uninsured children having poorer access to specialty care, compared to insured children (3).

Federal and State health insurance programs are intentionally designed for children and adults who are documented and lawfully residing in the United States (4). These programs do not make provision for undocumented families, the majority of whom are immigrant families. This increases the likelihood that children in these families will seek care in emergency care settings (5–9). The authors are all too familiar with children presenting to the emergency room with kidney failure and a late diagnosis of kidney disease. In nearly all cases, these children have not been seen by a primary care physician in many years. The principal objective of this article is to enhance community awareness on the scope of health care disparities among children and young adults with chronic kidney disease (CKD). We shall explore the social, cultural, and economic determinants of inadequate access to optimal kidney health care services. We shall also discuss the role of lack of health insurance on the promotion of CKD and offer recommendations on meaningful strategies to improve access to kidney health services in this population.

## Impact of Lack of Insurance Coverage on Kidney Health

Lack of health insurance is detrimental to health and has been shown in adult studies to be associated with increased odds of dying (10). In children, a recent study examining the sociodemographic risk factors for pediatric acute kidney injury [AKI] showed that children who lacked insurance were more likely to be admitted to hospital with AKI (11). It is well established that children with AKI are at risk for the development of CKD later on. Disadvantaged socioeconomic status in early childhood, and by implication, a higher likelihood of being uninsured, translates to a higher risk of kidney disease and end stage kidney disease [ESKD] in later life (12). Lack of insurance means that children are more likely to experience delays in seeking medical care and a higher likelihood of presentation to the emergency department for needed healthcare services (13). Anecdotal reports suggest that children with kidney disease who do not have health insurance coverage often present to the emergency department in kidney failure. Adult literature is robust with reports of patients with kidney failure depending on the emergency department for chronic hemodialysis services and the resultant implications on cost and mortality (14–16). Moreover, the higher cost of care in the emergency department translates to higher individual and national healthcare costs which drives poverty at the individual level and promotes national disparities in healthcare delivery (17, 18). Health problems associated with lack of insurance in childhood could lead to delays in the attainment of developmental milestones, which impacts productivity later in life.

## Cost Implications of CKD Care

Patients with CKD have extensive healthcare needs and significant financial burdens (19, 20). While it is difficult to accurately estimate the cost of care of non-dialysis dependent CKD, expenses associated with dialysis and kidney transplant services are traceable and enormous, and without insurance coverage, patients are likely to suffer serious adverse outcomes. A recent retrospective analysis of children undergoing conventional hemodialysis showed mean monthly cost estimates of $3,500, adding up to $87,000 in 40 months. For children who have undergone kidney transplantation, mean monthly costs were estimated at $1,900, adding up to $48,000 in 50 months (21). The 2021 United States Renal Data System (USRDS) annual report provides information on spending associated with pre-dialysis care. For commercially insured children, the cost of caring for children with CKD was $20,764 per person per year (PPPY), compared to $2,092 for children without CKD (19). These estimates do not account for self-pay and other forms of payment for services. In 2018, the USRDS estimates of the Medicare spending for beneficiaries of non-dialysis dependent CKD younger than 66 years exceeded $ 11 billion. For all patients with ESKD, a total spending estimate of $ 49 billion was reported for the same year (19).

Although Medicare pays for dialysis and transplant services for children with ESKD, coverage is dependent upon meeting certain eligibility criteria. This includes being the dependent child of a parent who is eligible for social security benefits, who has worked the required time under social security, or who is a government employee and by implication, lawfully residing in the United States. Until recently, coverage for children who received a kidney transplant ended after 36 months, leaving families and patients with the financial burden of paying for vital immunosuppressive medications. However, in December 2020, the law was changed to provide lifetime Medicare coverage for post kidney transplant immunosuppressive coverage (22).

The costs associated with CKD care may vary depending on the state and the healthcare facility where treatment is received. For patients who do not have insurance coverage, such as undocumented patients, public hospitals and emergency departments become the only access to healthcare services, and costs associated with these services are high (23). While some State Medicaid programs extend coverage to uninsured and undocumented individuals, many States do not. Currently, California, Illinois, Oregon, Massachusetts, New York, and Washington States extend healthcare coverage using state funds to income-eligible children regardless of their immigration status (24, 25).

As a result of the huge costs associated with CKD care, the majority of uninsured and poor patients are less likely to receive crucial pre-dialysis care, presenting late with severe azotemia, requiring urgent initiation of dialysis (23, 26). Moreover, available data shows that immigrant, black and Hispanic children are more likely to receive hemodialysis as a chronic dialysis modality, as compared to their white counterparts (19, 27, 28). Hemodialysis is associated with a lower quality of life in both children and adults compared to peritoneal dialysis (29, 30). Furthermore, uninsured patients are often not eligible for kidney transplantation due to a lack of funding, which means a longer time on dialysis and an increased risk for morbidity and mortality (31). Moreover, when uninsured children receive kidney transplantation out of compassionate care, payment for immunosuppressive medications becomes a challenge, predisposing them to premature graft loss (32, 33).

## Structural Barriers to Accessing Kidney Services

**Figure 1:**
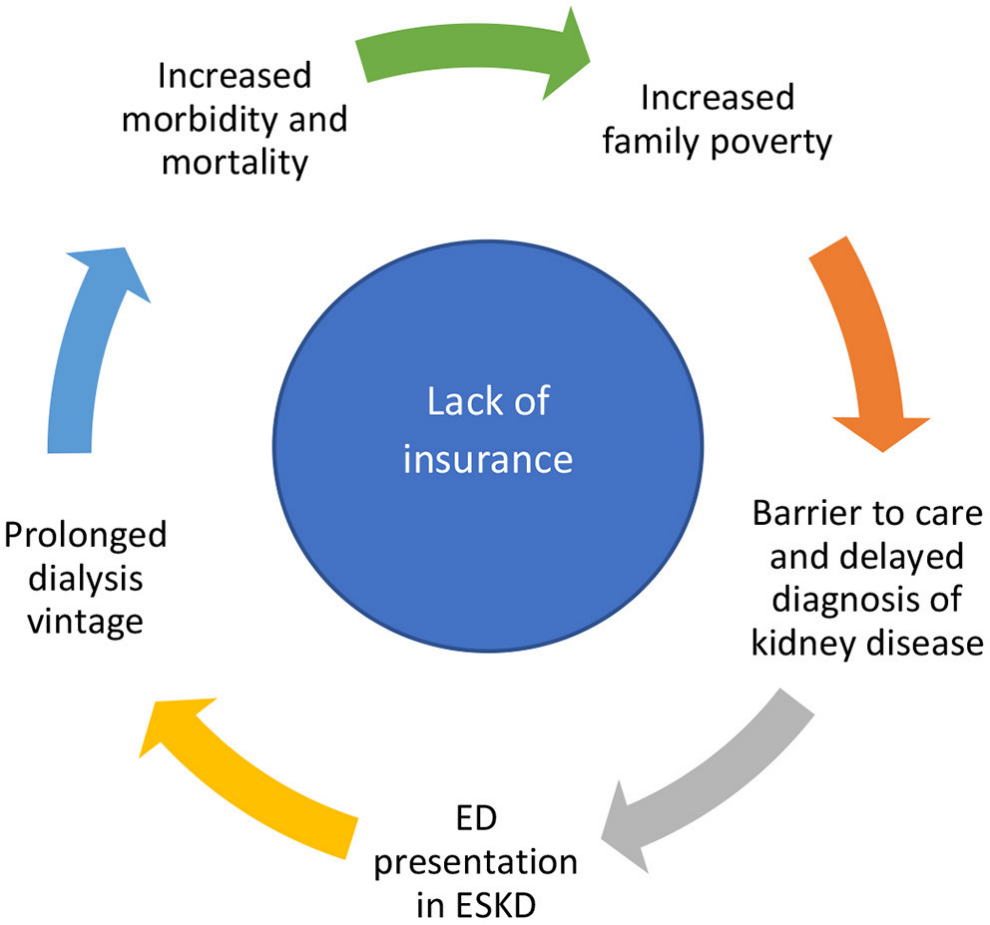
The circle of burden of the uninsured with chronic kidney disease. ED, emergency department; ESKD, end stage kidney disease.

Multiple structural barriers exist in the American political, socioeconomic and healthcare systems that hinder access to optimal healthcare (Table 1; Figure 1). We shall discuss the permissive role of these factors in the promotion of health disparity and CKD, as well as the adverse consequences on community life.

**Table 1:** Structural barriers to accessing healthcare services.

**Structural barriers**	**Comment**
Policy mandates	Federal and state insurance programs designed only for documented and legal residents
Lower educational attainments	This is associated with higher odds of being uninsured
Poor health literacy	This is associated with higher odds of being uninsured
Immigration status	Undocumented immigrants less likely to have insurance coverage
Language disparities	Immigrants often not proficient in English, predisposing to inefficient patient-provider communication
Anti-immigrant rhetoric	This instills fear and anxiety in undocumented immigrants and hinders healthcare seeking behavior
Structural racism and inequalities	Lower socioeconomic status and racism linked to higher rates of lack of insurance coverage

### Health Disparities and CKD in Marginalized Populations

Health disparity occurs in the context of systematic exclusion from adequate access to essential social and clinical services that are often based on racial, ethnic, socioeconomic, political, and religious differences. Other discriminatory factors of historical importance are cognitive dysfunction, physical disability, immigration status, rural geographical location, a younger or older age group, and gender identity or sexual orientation. As a result of expensive but required healthcare services, disparities in the treatment of CKD are particularly profound in marginalized communities. Genetic predisposition (as in APOL1 mutation) and socioeconomic disadvantage may account for the greater risk of developing CKD in Blacks compared with the White population (34, 35). Trend analysis between the year 2005–2014 showed a greater prevalence of diabetic CKD in Blacks and Hispanics compared to the White population (36). Moreover, poverty often leads to poor dietary choices which in turn promotes exogenous obesity and diabetic mellitus (37, 38). The latter is a leading cause of dialysis-dependent CKD in the USA. In a recent report, uninsured children were shown to be more likely to suffer AKI when compared to children with any health insurance (11). Furthermore, apart from promoting socio-economic deprivation, discriminatory housing policies may increase family exposure to environmental kidney toxins such as lead, mercury, cadmium, and arsenic heavy metals (39). Pregnancy may be complicated by adverse psychosocial and biological events including poor prenatal care, nutritional insufficiency, and premature delivery (leading to low nephron mass) that may produce permanent impairment in structural or functional programming of the fetal organs and result in the later development of cardiorenal morbidities (12, 40).

### Limitation of Access to Kidney Subspecialists

Unfavorable health insurance policy limits access of the socially disadvantaged to kidney subspecialists. Available evidence shows that marginalized population groups (including Blacks, Hispanics, and Asians, some of whom are undocumented) are less likely to receive kidney care in the last 12 months preceding the initiation of chronic dialysis due to lack of insurance coverage (41). Even when they have insurance coverage, the majority of these patients depend on primary care providers (PCP) for kidney-related care as a result of limited access to nephrologists. Unfortunately, there is often suboptimal quality of CKD education during encounters with PCP in the localities in which marginalized populations live (42–44). It is, therefore, necessary to work with community PCP to enhance their knowledge about the nature and complications associated with kidney disease. Our efforts should focus on preventative strategies such as avoiding the use of nephrotoxic medications as much as possible, prompt assessment and treatment of dehydration and infections. Early diagnosis and prompt therapeutic interventions to address morbidity in children living with CKD could also help preserve their kidney function and delay progression to ESKD. In rural areas with limited access to healthcare services, it may be necessary for PCP to share clinical responsibility with kidney specialists who are frequently located in urban environments. The recently available telemedicine resources may facilitate such engagements.

### Health Insurance Coverage

Although lack of health insurance coverage is the single most important barrier to accessing healthcare services, there are other salient contributory factors. The cost of healthcare services is often prohibitive and is mostly unaffordable for low-income families. Insurance plans are offered through a variety of programs including federal coverage through Medicaid and Medicare, employer-based plans, the Veteran's health administration and free- market plans. An understanding of this insurance structure takes concerted efforts and provision of quality education. A profile of the uninsured shows lower educational attainment, poor health literacy, and poverty (1, 45). These adverse social characteristics may be particularly profound among marginalized populations as depicted in Figure 1 (46). The significance of adequate health insurance coverage was demonstrated by a study that examined the impact of the Medicaid expansion program on the risk of kidney disease (47). There was a 3% reduction in the incidence of CKD among young adults in the first 3 years in the States that adopted Medicaid expansion in the USA. The largest benefit of this insurance practice was observed in the non-Hispanic White population. Similar studies looking at the impact of Medicaid expansion have shown improved access to kidney health services (48). The number of children and young adults covered by health insurance has also improved with Medicaid expansion (49, 50). Despite these gains in outcomes, universal health insurance coverage may not be enough to eradicate the disparity in the care of CKD. For instance, although the health insurance policy provided to the United States veterans regardless of ethnicity or social background helped improve access to renal physicians, it did not succeed in erasing the disparities and higher incidence of advanced kidney disease in the marginalized populations (51). Policies targeted at improving health literacy, and eradicating poverty and racism are also necessary.

### Public Charge Policy and Impact on Children's Healthcare Coverage

In October 2018, the Trump administration expanded the definition of “public charge” (defined as an immigrant who has received one or more public benefits for more than 12 months within any 36-month period) to include any immigrant who was likely to receive public benefits in the future. If they were found likely to receive public benefit in the future, they were excluded from getting a green card or visa to the United States. This rule posed a threat to healthcare coverage for immigrants and their families (52). A simulation of the potential harms that would be caused by this rule showed that millions of children would lose healthcare coverage including many with specific medical needs that, if left untreated, could contribute to child deaths and future disability (53). Thankfully, the new public charge regulation was reversed by the Biden administration, in March, 2021, protecting many immigrant children from losing health insurance coverage.

### Illegal Immigration

Globalization in the twenty-first century has seen an exponential rise in the transnational migration of people, resulting in changes in socio-economic, political, cultural, and healthcare structures of various geographical regions around the world. Immigrant families are often of ethnic minority and lower socioeconomic status and may therefore endure discriminatory health care policies (54–58). Illegal immigration status has been linked to a lack of insurance policy and a limited ability to access a comprehensive healthcare service (59, 60). Immigrant children are more likely to be uninsured and lack access to healthcare, which increases the likelihood of delays in the diagnosis of their medical conditions, including kidney disease (1, 61). Language disparities also constitute a barrier to accessing healthcare services amongst non-English speaking immigrant families (Table 1). Such ineffective communication invariably compromises the quality of health care delivery, ability to adhere to medical recommendations, and even return for follow-up visits (61–64).

Public anti-immigrant rhetoric limits access to healthcare by instilling fear of deportation, anxiety, and even depression in undocumented immigrants. This scenario frequently results in significant delays in seeking and receiving healthcare services, greater risk for more rapid progression of the disease and an earlier need for initiation of renal replacement therapy in patients with kidney disease (65).

### Socioeconomic Status

Lower socioeconomic status is a determinant of health problems and has been linked to increased morbidity and mortality, reduced access to healthcare, and poorer health outcomes. Moreover, the absence of medical homes, sedentary lifestyles and poor nutritional quality, invariably promote exogenous obesity, hyperlipidemia, hypertension, diabetes mellitus, and ultimately cardio-renal morbidity (66, 67). A recent meta-analysis showed there was a predictive relationship between a low socioeconomic status and a decline in glomerular filtration rate (GFR) (OR = 1.4; 95% CI = 1.2–1.6), degree of albuminuria (OR = 1.5; 95% CI = 1.2–1.8) and ESKD (OR = 1.5; 95% CI = 1.4–1.7) (68). In another study, families with housing insecurity were shown to have a three-fold greater likelihood of developing incidental kidney disease (69). With reduced access to healthcare services, these individuals are more likely to experience delays in seeking medical intervention.

A remarkable example of how a reversal of adverse social environment (enhanced nutrition and physical exercise) could produce a favorable clinical outcome was demonstrated in the indigenous American community. Introduction of comprehensive indigenous health services to prevent and optimize the care of diabetes mellitus resulted in a 50% reduction in the incidence of ESKD. This intervention led to a decline in the prevalence of ESKD from the highest rate observed in 1996 to a level below that of Black and Hispanic populations by the year 2013 (70).

## Summary and Policy Recommendations

In summary, there is need for policymakers to re-examine the implications of leaving out certain population groups from insurance coverage. Lack of health insurance may be associated with significant cost implications and put a strain on healthcare expenditures (71). Studies on emergency department (ED) utilization in children show that uninsured children, some of whom may have CKD, are more likely to depend on the ED for their healthcare needs (13). Emergency care services are invariably expensive and contribute to greater financial burden in the healthcare system. Moreover, children with CKD who rely on ED care are more likely to present in severe kidney failure. This late presentation often means that patients are sicker and need more intense and expensive care. A comparison of scheduled vs. emergency-only dialysis in adult undocumented immigrants with ESKD has shown substantial cost savings when dialysis is provided in a planned and scheduled manner (15). Moreover, extending insurance coverage to undocumented immigrants and those who cannot afford to purchase insurance might lead to state and federal cost savings and improve the health of all U.S residents (72–74). Withholding kidney transplantation in children and young adults on dialysis due to an unfunded status is not only unethical but also increases morbidity and mortality. Similarly, providing chronic hemodialysis via a catheter instead of a fistula is fraught with access complications that increase the economic burden of dialysis care.

The root cause of health disparities in CKD in the United States is complex and includes modifiable social determinants such as poverty, poor nutrition, and racial segregation. Policymakers need to address the deficit in essential social programs by providing economic empowerment, improved educational standard, affordable quality food, access to a safe neighborhood, optimal recreational program, and reduction of environmental pollution. In this regard, the recent award of a $500,000 grant by the U.S. Department of Health and Human Services Office of Minority Health to support the collection of health data on community social determinants to address health disparities is a step in the right direction. A universal health insurance policy will minimize delay in seeking medical intervention, reduce the use of vascular catheters, decreases hospitalization rate, promotes the utilization of peritoneal dialysis, and enhance the opportunity for pre-emptive kidney transplantation. The summative effect is a substantial saving in health care cost, increase in human productivity, and a better quality of life for the citizens.

## Author Contributions

Both authors listed have made a substantial, direct, and intellectual contribution to the work and approved it for publication.

## Conflict of Interest

The authors declare that the research was conducted in the absence of any commercial or financial relationships that could be construed as a potential conflict of interest.

## Publisher's Note

All claims expressed in this article are solely those of the authors and do not necessarily represent those of their affiliated organizations, or those of the publisher, the editors and the reviewers. Any product that may be evaluated in this article, or claim that may be made by its manufacturer, is not guaranteed or endorsed by the publisher.
